# Salicylic acid signaling inhibits apoplastic reactive oxygen species signaling

**DOI:** 10.1186/1471-2229-14-155

**Published:** 2014-06-04

**Authors:** Enjun Xu, Mikael Brosché

**Affiliations:** 1 Division of Plant Biology, Department of Biosciences, University of Helsinki, P.O. Box 65 (Viikinkaari 1), FI-00014 Helsinki, Finland; 2 Institute of Technology, University of Tartu, Nooruse 1, Tartu 50411, Estonia

**Keywords:** Cell death, Ethylene, Gene expression, Jasmonic acid, Reactive oxygen species, Salicylic acid

## Abstract

**Background:**

Reactive oxygen species (ROS) are used by plants as signaling molecules during stress and development. Given the amount of possible challenges a plant face from their environment, plants need to activate and prioritize between potentially conflicting defense signaling pathways. Until recently, most studies on signal interactions have focused on phytohormone interaction, such as the antagonistic relationship between salicylic acid (SA)-jasmonic acid and cytokinin-auxin.

**Results:**

In this study, we report an antagonistic interaction between SA signaling and apoplastic ROS signaling. Treatment with ozone (O_3_) leads to a ROS burst in the apoplast and induces extensive changes in gene expression and elevation of defense hormones. However, *Arabidopsis thaliana dnd1* (*defense no death1*) exhibited an attenuated response to O_3_. In addition, the *dnd1* mutant displayed constitutive expression of defense genes and spontaneous cell death. To determine the exact process which blocks the apoplastic ROS signaling, double and triple mutants involved in various signaling pathway were generated in *dnd1* background. Simultaneous elimination of SA-dependent and SA-independent signaling components from *dnd1* restored its responsiveness to O_3_. Conversely, pre-treatment of plants with SA or using mutants that constitutively activate SA signaling led to an attenuation of changes in gene expression elicited by O_3_.

**Conclusions:**

Based upon these findings, we conclude that plants are able to prioritize the response between ROS and SA via an antagonistic action of SA and SA signaling on apoplastic ROS signaling.

## Background

As sessile organisms, plants have evolved a highly sophisticated and elaborate signaling network to respond and adapt to various biotic and abiotic stresses. To precisely respond to diverse stimuli in different tissues or developmental stages, the defense signaling network must be orchestrated within a larger physiological and developmental context. Numerous data from large scale transcriptome profiling analysis strongly support the existence of regulatory interactions and coordination between signaling networks, rather than linear pathways [1,2]. To some extent the signaling components of this intricate network to biotic and abiotic stresses are universal [3,4]. Comparing multiple gene expression experiments performed on the Affymetrix ATH1 platform has identified a universal stress response transcriptome [5]. In addition to a general stress response, there are also several studies that indicate that plants are able to prioritize between different stresses and that a combination of stresses leads to unique gene expression profiles [6-10]. Execution of an appropriate defense response is linked to multiple interacting components, including a rapid and transient Reactive Oxygen Species (ROS) burst, altered cytoplasmic and chloroplastic Ca^2+^ transients, plant hormones including salicylic acid (SA), jasmonic acid (JA), abscisic acid (ABA), ethylene, and transcriptional reprogramming [11-13].

Activation of a ROS burst is a common response to both biotic and abiotic stress [14,15]. In addition, ROS are signaling molecules involved in control and regulation of other biological processes, such as aging, cell death, and development [16,17]. Exposure of plants with the gaseous ROS ozone (O_3_) triggers an apoplastic ROS production, which is similar to the ROS burst observed after pathogen infection and activation of cell wall peroxidases and NADPH oxidases [14,18,19]. Extensive comparisons of altered gene expression profiles from *Arabidopsis thaliana* elicited by O_3_ and other abiotic and biotic stresses indicate a high degree of overlap between O_3_ and treatment with a bacterial microbe associated molecular pattern (MAMP) flg22 [13,20-22]. One of the earliest responses elicited by flg22 treatment is an apoplastic ROS burst [23,24], thus providing a mechanistic link for the similarity between gene expression changes elicited by O_3_ and flg22. Apoplastic ROS are also regulators of cell death through interplay with several other signaling pathways, including SA and JA/ethylene signaling pathways [25].

SA, JA, and ethylene are involved in many aspects of defense signaling and numerous studies have investigated the interaction between these hormones [26]. It is generally believed that antagonism between SA and JA allows plants to prioritize the defense between biotrophic or necrotrophic pathogens and insects. SA antagonism of JA signaling is a robust response observed both when plants are infected with different pathogens [27]; and when plants are directly treated with hormones [28]. Regulators of the SA-JA antagonism include the SA receptor/transcriptional co-activator NPR1 and the transcription factor ORA59 [29,30]. Several additional signals directly or indirectly interplay with SA to promote defense response [31]. Early in 1990s, SA level and ROS (e.g. H_2_O_2_) production were found to be closely connected [32]. Both elevated endogenous SA and application of exogenous SA in *Arabidopsis* and tobacco are accompanied by increased ROS (H_2_O_2_ and O_2_^-^) production [33-36], indicating the existence of a positive feedback amplification loop with SA and ROS as central players. However, continuous defense signal amplification would waste energy and indicate that coordination of SA-dependent and independent signaling components with ROS signaling are of central importance to provide an appropriate defense response.

Lesion mimic mutants that display spontaneous cell death have been extensively used to study the regulation of cell death [37]. In addition to misregulated cell death they often have other phenotypes including dwarfism, constitutively higher accumulation of SA and enhanced pathogen resistance [38,39]. Some of them show accumulation of ROS (H_2_O_2_ and O_2_^-^) in or around the lesion area [40], which make lesion mimic mutants a powerful tool to investigate the relationship between ROS and SA. In genetic analysis, production of SA can be reduced by the mutation *sid2,* which is defective in the main biosynthesis pathway (ISOCHORISMATE SYNTHASE1, ICS1), or by expression of a bacterial SA degrading enzyme *NahG*. In several lesion mimic mutants, including *acd6*, *acd11* and *lht1* expression of *NahG* abolishes cell death [41-43]. Given the importance of SA in defense signaling it is not surprising that several other regulators working in parallel with SA signaling, or affecting SA accumulation, have been identified through various screens including suppression of lesion mimic phenotypes [44-46]. These regulators include ENHANCED DISEASE SUSCEPTIBILITY1 (EDS1), AG2-LIKE DEFENSE RESPONSE PROTEIN1 (ALD1) and FLAVIN-DEPENDENT MONOXYGENASE1 (FMO1) which regulate cell death and defense responses [46-49]. Like *SID2*, *ALD1* and *FMO1* are necessary for systemic accumulation of SA and downstream signaling after pathogen infection [49,50]. Furthermore, a chloroplastic derived O_2_^-^ signal can be processed by EDS1 to control SA-dependent H_2_O_2_ accumulation as part of a mechanism limiting cell death [51].

Elevation of cytosolic Ca^2+^ and production of ROS are among the earliest events after initiation of stress responses [52]. Many studies have explored the role of CNGC2 (CYCLIC NUCLEOTIDE GATED CHANNEL2) in regulation of Ca^2+^ fluxes across the plasma membrane and its contribution to signaling in the context of immunity [53,54], senescence [55], heat stress [56], and pollen growth [57]. Null mutation of CNGC2 was first isolated as *defense no death1* (*dnd1*), a mutant which exhibits a lesion mimic phenotype which is dependent on growth conditions [58], increased accumulation of SA and constitutive defense activation [59], and altered Ca^2+^ transport [60]. Studies conducted in this mutant indicate that the influx of Ca^2+^ is associated with the pleiotropic phenotype; however, the precise mechanism with regards to Ca^2+^ signaling is still elusive. Furthermore, whereas in wildtype O_3_ strongly alters transcript levels for many defense genes, in *dnd1* this response is blocked [20]. Due to the pleiotropic phenotype of *dnd1* it is far from straight forward to pinpoint the exact process which blocks the apoplastic ROS signal initiated by O_3_ treatment. In this study we investigate through genetic analysis the relationship between *dnd1*, the hormones SA, JA and ethylene, and apoplastic ROS signaling in the regulation of defense gene expression and cell death. In particular, we identify a novel antagonistic interplay between SA and apoplastic ROS signaling that may confer a high degree of responsiveness of plant responses to a fluctuating environment.

## Results

### The *dnd1* mutant displays constitutive expression of defense genes

**Figure 1:**
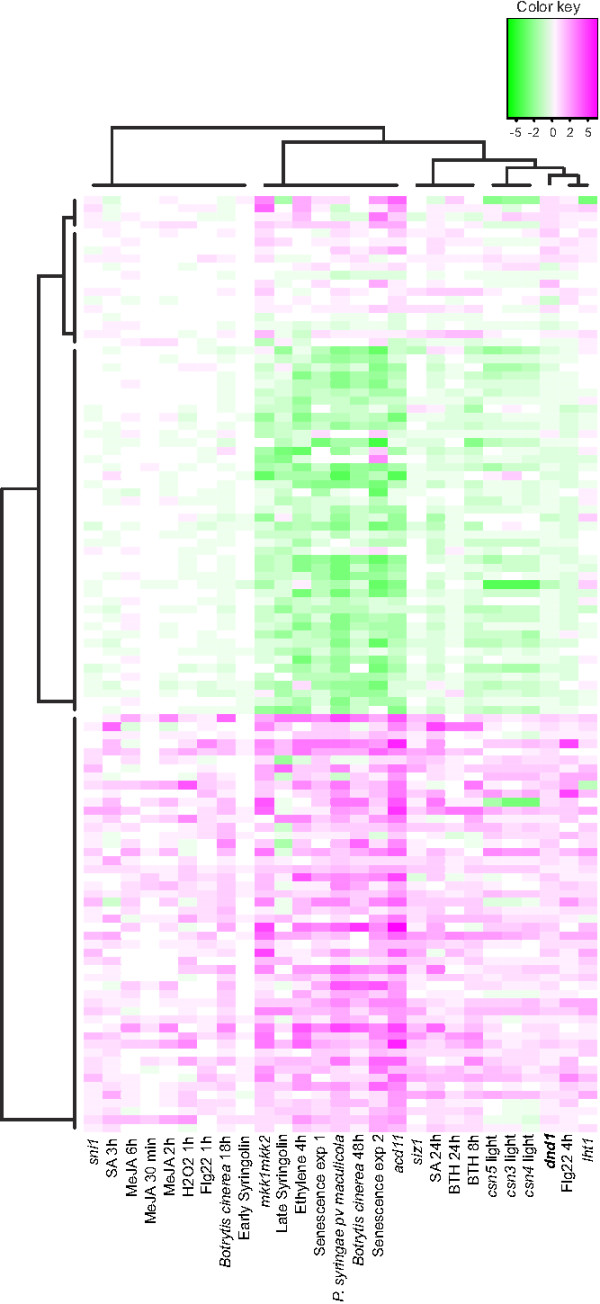
**Cluster analysis of *dnd1*-regulated genes.** Cluster analysis of transcripts with altered expression in *dnd1*, in comparison with lesion mimic mutants, constitutive defense mutants, hormone treatments, senescence and biotic and abiotic stresses. Bayesian hierarchal clustering of genes is shown in plants subject to different stress treatments compared with control condition, or in mutant versus wildtype. Values are mean of log2 ratio of the treatment and control expressions. Magenta and green indicate increased and decreased expression compared with untreated or wild-type plants, respectively.

The *dnd1* mutant displays constitutively elevated concentration of SA and increased expression of SA induced and defense related genes [61,62]. However, the phenotypes of *dnd1*, including the appearance of spontaneous cell death, is influenced by growth conditions [58]. Hence, we performed DNA microarray analysis on wildtype and *dnd1* from our growth conditions using six biological repeats (see Methods). 69 genes had increased expression and 49 genes had decreased expression (Additional file 1). The annotations for many of these genes in the TAIR database (http://www.arabidopsis.org/) indicated a function in plant defense responses. To systematically evaluate the role of these genes in plant stress responses a Bayesian hierarchal clustering was made with *dnd1* and experiments performed on the Affymetrix ATH1 chip obtained from public databases (Figure 1). These experiments were selected to include pathogen infection, mutants that display constitutive defense activation or spontaneous cell death, and the stress hormones SA, its analog benzo (1,2,3) thiadiazole-7-carbothioc acid S-methyl ester (BTH), methyl-jasmonic acid (MeJA) and ethylene (see Methods for a complete list of the experiments used). The genes with increased or decreased expression in *dnd1* were consistently regulated in the similar direction by flg22, late SA and BTH treatment, in mutants undergoing cell death *mkk1mkk2*, *acd11*, *csn3*, *csn4* and *csn5* and in the constitutive defense mutants *siz1* and *lht1*. Cell death in these mutants are initiated via different mechanisms: *mkk1mkk2* is defective in two MAP kinase kinases, *acd11* lacks a ceramide-1-phosphate transfer protein, *csn3*, *csn4* and *csn5* lack different subunits in the COP9 signalosome – a regulator of protein degradation, *siz1* lacks a SUMO E3 ligase and *lht1*lacks a lysine-histidine transporter [43,63-66]. Despite different biological mechanisms being altered in these mutants, they displayed a common set of misregulated genes; this could indicate that cell death is executed through a common mechanism. We conclude that *dnd1* in our growth conditions displayed a constitutive activated defense gene expression profile, similar to other mutants of this class [37].

### Mutants with constitutive defense activation are defective in ROS signaling

**Figure 2:**
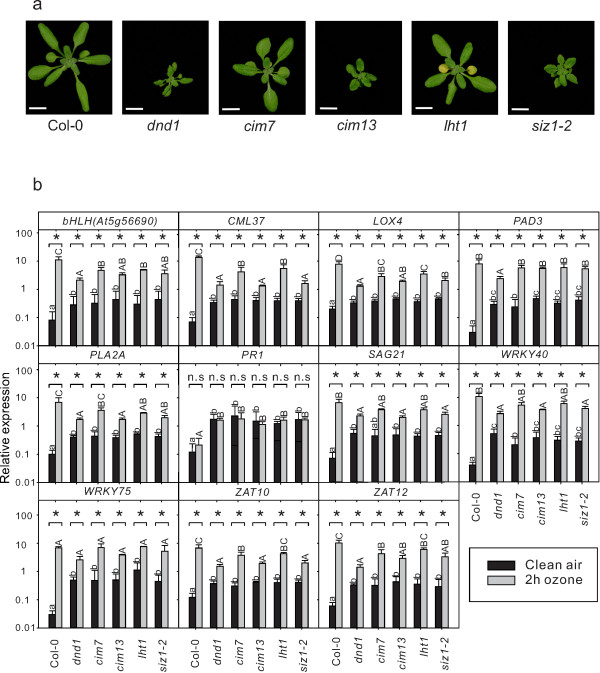
**Arabidopsis mutants with constitutive defense activation are deficient in ROS signaling. a**, Morphology of three weeks old *Arabidopsis* constitutive defense/lesion mimic mutants *dnd1*, *cim7 (constitutive immunity 7)*, *cim13 (constitutive immunity 13)*, *lht1 (lysine histidine transporter1)* and *siz1*-*2* (scale bar 1 cm). **b**, Relative expression of marker genes in clear air and after two hours of 350 nL L^-1^ O_3_ were analyzed with qPCR in constitutive defense/lesion mimic mutants and wildtype. Values are the mean ± SD of four biological replicates. Statistical significance (P < 0.05) was calculated on log2 transformed data by Two-way ANOVA with Tukey-test. (a, b, c represent comparison of different genotypes in clean air; A, B, C represent comparison of different genotypes in O_3_ treatment; * and n.s represent comparison between clean air and O_3_ treatment in each genotype). *bHLH*(At5g56690): basic helix-loop-helix transcription factor; *CML37*: *CALMODULIN LIKE 37*; *LOX4*: *LIPOXYGENASE 4*; *PAD3*: *PHYTOALEXIN DEFICIENT 3*; *PLA2A*: *PHOSPHOLIPASE A 2A*; *PR1*: *PATHOGENESIS-RELATED GENE 1*; *SAG21*: *SENESCENCE-ASSOCIATED GENE 21*; *WRKY40*: *WRKY DNA-BINDING PROTEIN 40*; *WRKY75*: *WRKY DNA-BINDING PROTEIN 75*; *ZAT10*: *SALT TOLERANCE ZINC FINGER 10*; *ZAT12*: *SALT TOLERANCE ZINC FINGER 12*.

Treatment of plants with O_3_ generates a precise burst of apoplastic ROS and is a convenient tool to study the role of ROS in regulation of defense gene expression [21]. Previous analysis of defense marker genes in *dnd1* treated with O_3_ indicated that this mutant had an attenuated response [20]. However, the role of the *DND1*/*CNGC2* protein in apoplastic ROS signaling is an open question, since the pleiotropic phenotype of *dnd1*, including high SA concentration and constitutive activation of defense genes could be the source of altered ROS signaling and not the lack of Ca^2+^ transport from removal of *DND1*/*CNGC2*. To explore this question, *dnd1* and several other mutants with increased SA concentration and constitutive defense gene expression, *cim7*, *cim13*, *lht1* and *siz1*-2 [43,66,67] were tested in gene expression analysis using real time reverse transcriptase quantitative PCR (qPCR) with marker genes selected from the *dnd1* array analysis (Additional file 1) and previous O_3_ gene expression analysis [20,21,68]. The constitutive defense mutants were selected to include both dwarfed mutants and mutants with more wildtype morphology (Figure 2a).

Consistent with previous characterization of these mutants as constitutive defense mutants, a majority of the marker genes, including *PAD3*, *SAG21*, *WRKY40* and *WRKY75*, had increased expression in the mutants as compared to Col-0 in control conditions (Figure 2b; note the logarithmic scale). A two hour O_3_ treatment led to strong induction of the defense genes in Col-0, whereas the effect of O_3_ was attenuated in all of the constitutive defense mutants, which was more pronounced in *dnd1* (Figure 2b). We conclude that constitutive activation of defense signaling in several different mutants interfere with the plants ability to properly respond to a ROS signal from the apoplast.

### SA signaling inhibits apopastic ROS signaling

**Figure 3:**
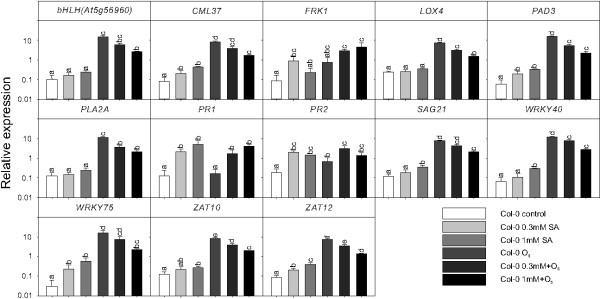
**Exogenous SA treatment attenuates the effect of apoplastic ROS signaling.** Relative expression of marker genes in response to exogenous 0.3 and 1 mM SA pretreatment (24 hours), two hours of 350 nL L^-1^ O_3_, or the combined treatment were analyzed by qPCR in wildtype Col-0. Values are the mean ± SD of three biological replicates. Statistical significance (P < 0.05) was calculated on log2 transformed data by One-way ANOVA with Tukey-test. (a, b, c, d, e, f represent comparison of different treatments in Col-0).

The results in Figure 2 indicate that SA signaling has the capacity to interfere with apoplastic ROS signaling. To directly test the role of SA, plants were treated with 0.3 or 1 mM SA 24 hours before a two hours O_3_ exposure (Figure 3). Treatment with SA alone increased the expression of the classical SA marker genes *PR1* and *PR2*. Furthermore, several of the other marker genes in this study were also regulated by SA, including *CML37*, *PAD3*, *SAG21*, *WRKY40*, *WRKY75*, *ZAT10* and *ZAT12*. Strikingly, SA pre-treatment at both concentrations significantly reduced the response to subsequent treatment with ozone for all marker genes except *PR1* and *PR2* (Figure 3). In an attempt to also identify marker genes with an opposite behavior (i.e. additive effect of combined SA and ozone treatment, rather than an antagonistic effect), we tested the expression of *FRK1*, a flg22 responsive gene [69]. In contrast to all other genes tested, expression of *FRK1* was synergistically increased by the combined SA and ozone treatment.

**Figure 4:**
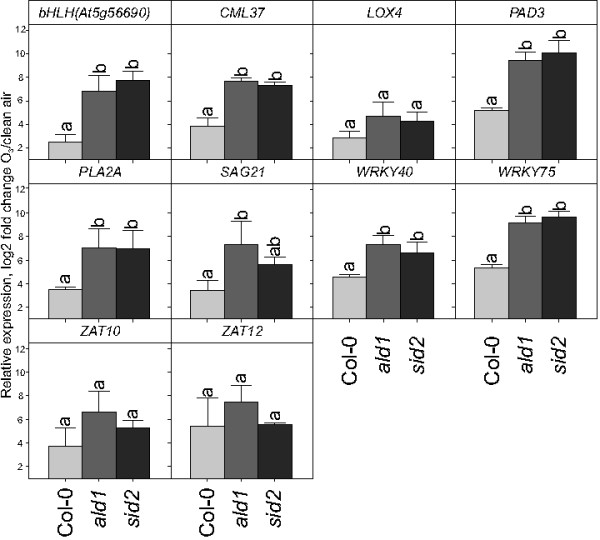
**Reduction of endogenous SA increases the effect of apoplastic ROS signaling.** Relative expression of marker genes in clean air and after two hours of 350 nL L^-1^ O_3_ were analyzed with qPCR. Values are mean of log2 fold change ± SD of three biological replicates. Statistical significance (P < 0.05) was calculated by One-way ANOVA with Tukey-test. (a,b represent comparison among different genotypes).

To further explore whether low endogenous SA level alter apoplastic ROS signaling, the SA biosynthesis deficient *sid2* and the low SA accumulation mutant *ald1* were used [50,70]. In both mutants O_3_ treatment led to a stronger induction of most marker genes than observed in wt plants (Figure 4). We conclude that there exists an inhibition by SA on apoplastic ROS signaling in transcriptional activation of defense related genes.

### Elimination of SA in *dnd1* partially restores its response to apoplastic ROS

**Figure 5:**
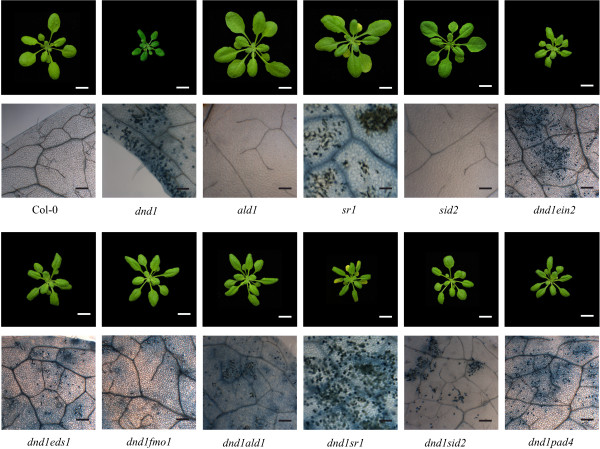
**SA-dependent and SA-independent signaling both contribute to development of cell death in *dnd1*.** Cell death of three weeks old plants were visualized and microscopically examined by trypan blue staining. From three rosettes per genotype and staining, one fully expanded and representative leaf (not the oldest leaf) was used for figures. White scale bar 1 cm, black scale bar 200 μM.

**Figure 6:**
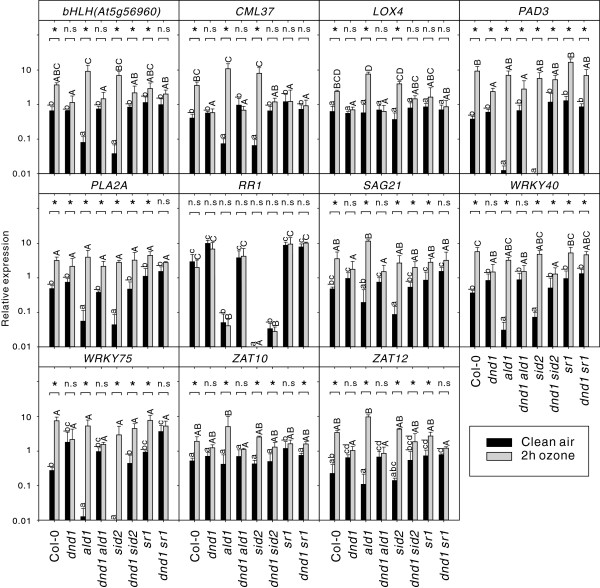
**Abolishing SA biosynthesis in *dnd1 *partially restores its response to apoplastic ROS signaling.** Relative expression of marker genes in clear air and after two hours of 350 nL L^-1^ O_3_ were analyzed with qPCR. Values are the mean ± SD of three biological replicates. Statistical significance (P < 0.05) was calculated on log2 transformed data by Two-way ANOVA with Tukey-test. (a, b, c, d represent comparison of different genotypes in clean air; A, B, C, D represent comparison of different genotypes in O_3_ treatment; * and n.s represent comparison between clean air and O_3_ treatment in each genotype).

Both ROS and SA are involved in defense signaling and regulation of cell death responses. Furthermore, elevated levels of ROS lead to SA accumulation and vice versa, which has been termed the oxidative cell death cycle [25]. However, deciphering all components involved in the complex ROS signaling network through a genetic analysis requires the use of mutants involved in different signaling pathways [71]. The *dnd1* mutant was crossed with various other mutants defective in different hormone signals, MAP kinases, transcription factors, ROS biosynthesis or mutants with a previously described role in cell death or defense against pathogens (Table 1). The extent of cell death in the double and triple mutants was examined with trypan blue staining (Figure 5 and Additional file 2). Of these double mutants, SA biosynthesis or SA signaling related genes *SID2*, *ALD1*, *EDS1* and *FMO1* reduced the amount of cell death and partially restored the altered leaf morphology of *dnd1* (Table1, Figure 5 and Additional file 2). In selected double mutants gene expression was tested after two hours O_3_ treatment (Figure 6). In *dnd1sid2* a partially restored response to O_3_ was observed (Figure 6). In contrast, although loss of *ald1* in *dnd1* background reduced the amount of cell death, it did not impact on the O_3_ induced gene expression profile (Figure 6).

**Table 1:** **Morphology and amount of cell death in *dnd1 *** **single, double and triple mutants**

**Genotype**	**Cell death**	**Size of the rossette**	**Involved in**	**Leaf shape**	**Reference to single mutant**
Col-0		+++++++			
*dnd1*	++++	++++	SA/Defense/Ca^2+^ channel	Slim and curled leaves	[61]
*dnd1 ein2*	++++	+++	ET	*	[72]
*dnd1 etr1-1*	+++++	++	ET	*	[73]
*dnd1 mpk3*	++++	++++	MAP kinase	*	[74]
*dnd1 mpk6*	++++	++++	MAP kinase	*	[74]
*dnd1 ibr5*	+++++	++++	MAP kinase phosphatase	More curled leaves than *dnd1*	[75]
*dnd1 rbohDrbohF*	+++++++	+	ROS biosynthesis	*	[76]
				**Note:** Highly dwarfed, seedless	
*dnd1 rbohD*	++++	++++	ROS biosynthesis	*	[76]
*dnd1 rbohF*	++++	++++	ROS biosynthesis	*	[76]
*dnd1 WRKY70*	++++	++++	Transcription factor	*	[77]
*dnd1 jin1/myc2*	+++++	++++	JA/Transcription factor	More curled leaves than *dnd1*	[78]
*dnd1 wrky25*	+++++	++++	Transcription factor	*	[79]
*dnd1 aos*	++++	++	JA biosynthesis	*	[80]
				**Note:** No trichomes, male sterile	
*dnd1 sid2*	+++	+++++	SA biosynthesis	Wider leaves than *dnd1*	[81]
*dnd1 npr1*	++++	++++	SA/Defense	Wider leaves than *dnd1*	[82]
				**Note:** Bleached leaves	
*dnd1 eds1*	+++	++++++	SA/Defense	*	[83]
*dnd1 pad4*	+++	+++++	SA/Defense	*	[84]
*dnd1 ald1*	+++	+++++	SA/Defense	*	[50]
*dnd1 fmo1*	++	++++++	SA/Defense	Leaf is less curled than *dnd1* single mutant	[47]
*dnd1 CBP60g*	+++++	+++	SA/Transcription factor	*	[85]
*dnd1 sr1/camta3*	++++++	+++	SA/Transcription factor	*	[86]
*dnd1 agb1gpa1*	+++	++++	G protein subunits	Round leaf shape	[87]
*dnd1 era1*	++++	+++	ABA	Wider leaves than *dnd1*	[88]
				**Note:** Slow growth, difficult to obtain seeds	
*dnd1 rar1-21*	++++	++++	R gene mediated proteins/Defense	*	[89]
*dnd1 acd5*	+++++++	+	SA/Defense	*	[90]
				**Note:** Few seeds	
*dnd1 sid2 ald1*	++	++++++	SA/Defense	Wider leaves than *dnd1*	
*dnd1 sid2 eds1*	++	++++++	SA/Defense	Wider leaves than *dnd1*	
*dnd1 aos sid2*	++++	++++	SA/JA/Defense	Wider leaves than *dnd1*	
				**Note:** No trichomes, male sterile	
*dnd1 sid2 pad4*	++	++++++	SA/Defense	Wider leaves than *dnd1*	
*dnd1 ald1 pad4*	++	++++++	SA/Defense	Wider leaves than *dnd1*	

**Figure 7:**
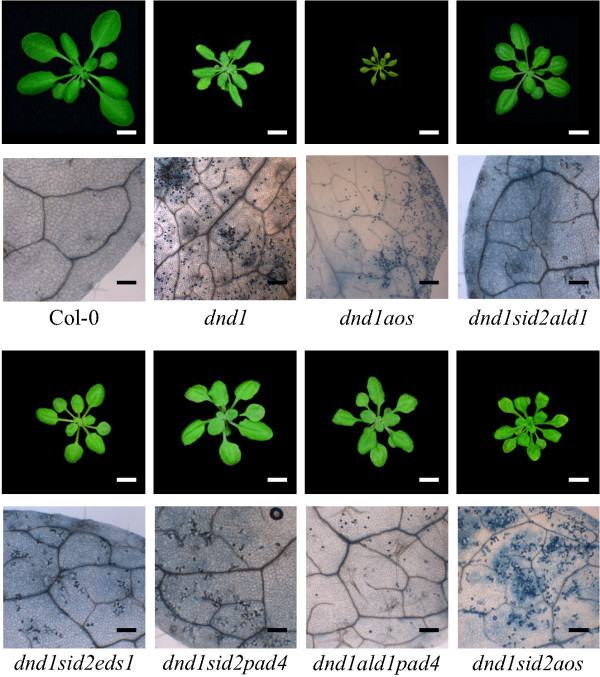
**SA-dependent and SA-independent signaling additively regulate development of cell death.** Cell death of three weeks old plants were visualized and microscopically examined by trypan blue staining. From three rosettes per genotype and staining, one fully expanded and representative leaf (not the oldest leaf) was used for figures. White scale bar 1 cm, black scale bar 200 μM.

+represent the relative extent of cell death or size of the rosette. Cell death was determined by trypan blue staining as in Figures 5 and 7.

*represent similar leaf shape as *dnd1* single mutant.

The name given to *dnd1*, defense no death, was based on its lack of pathogen induced cell death [58]. Numerous other mutants with spontaneous cell death and elevated levels of SA have been identified and includes *accelerated cell death 5* (*acd5*) and CALMODULIN BINDING TRANSCRIPTION ACTIVATOR (*CAMTA3/sr1*) [90,91]. To further explore if the cell death phenotype of *dnd1* was due to activation of similar signaling pathway as in other lesion mimic mutants, we introduced the *acd5* and *sr1* mutations into the *dnd1* background. The lesion and dwarfism phenotype in the resulting double mutants were severely enhanced, indicating that *dnd1* activated cell death in parallel pathways to *acd5* and *sr1* (Figure 5 and Additional file 2).

### Several SA dependent and independent regulators additively contribute to the attenuated apoplastic ROS response

**Figure 8:**
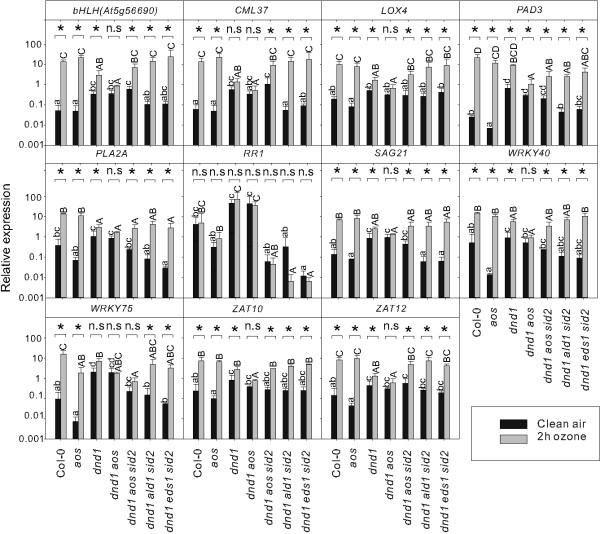
**Simultaneous inactivation of SA-dependent and SA-independent signaling pathways restore a normal apoplastic ROS response in *dnd1*.** Relative expression of marker genes in clear air and after two hours of 350 nL L^-1^ O_3_ were analyzed with qPCR in *dnd1*, *dnd1aos* and *dnd1* triple mutants. Values are the mean ± SD of three biological replicates. Statistical significance (P < 0.05) was calculated on log2 transformed data by Two-way ANOVA with Tukey-test. (a, b, c, d represent comparison of different genotypes in clean air; A, B, C, D represent comparison of different genotypes in O_3_ treatment; * and n.s represent comparison between clean air and O_3_ treatment in each genotype).

The results presented in Figure 5 and Additional file 2 demonstrated that SA biosynthesis and signaling regulators, such as *ALD1*, *PAD4*, and *EDS1* were important for spontaneous lesion formation since inactivation of either one of them partially rescued the *dnd1* spontaneous cell death phenotype. To further explore how these defense signaling regulators interplayed and contributed to the development of cell death and apoplastic ROS response, a number of combinations were made among these genes in *dnd1* background (Figure 7). The triple mutants *dnd1ald1sid2*, *dnd1eds1sid2*, *dnd1sid2pad4* and *dnd1ald1pad4* had less cell death and better growth than all double mutants (Table 1, Figures 5 and 7 and Additional file 2). To further investigate the role of the combination of these genes in relation to apoplastic ROS response, *dnd1ald1sid2* and *dnd1eds1sid2* were treated with two hours O_3_ and gene expression of selected marker genes tested with qPCR. Remarkably, inactivation of either *ald1* or *eds1* in *dnd1sid2* genetic background completely restored the O_3_ response in *dnd1* to the wild type (Figure 8). We conclude that several SA dependent and independent signaling pathways, mediated via ALD1 and EDS1, are co-activated in lesion formation and contribute to the attenuated apoplastic ROS signaling response in *dnd1*.

### JA signaling restricts lesion formation

Interplay between the hormones SA and JA optimizes the response to abiotic and biotic stresses [12,92]. In addition, the JA insensitive mutants *jar1* and *coi1* have previously been shown to be sensitive to O_3_[14,18,21]. To gain further insight into the role of JA in the *dnd1* pleiotropic phenotypes, a mutation that blocks JA biosynthesis [80], *allene oxide synthase* (*aos*) was introduced into *dnd1* and *dnd1sid2*. The *dnd1aos* double mutants showed severe dwarfism compared to *dnd1* single mutant (Figure 7 and Additional file 2). Similar to the *dnd1* single mutant, the *dnd1aos* double mutant had an attenuated gene expression response after two hours O_3_ treatment (Figure 8). Simultaneous mutations of both SA and JA signaling in *dnd1sid2aos* relieved the growth retardance of the *dnd1aos* double mutant, but induced more visible chlorosis than either *dnd1aos* or *dnd1sid2* double mutants (Figure 7). We conclude that JA has no major role in the attenuation of apoplastic ROS signaling, but is involved in regulation of plant development and cell death in the *dnd1* background.

### Constitutive activation of ethylene signaling does not impact on apoplastic ROS signaling

**Figure 9:**
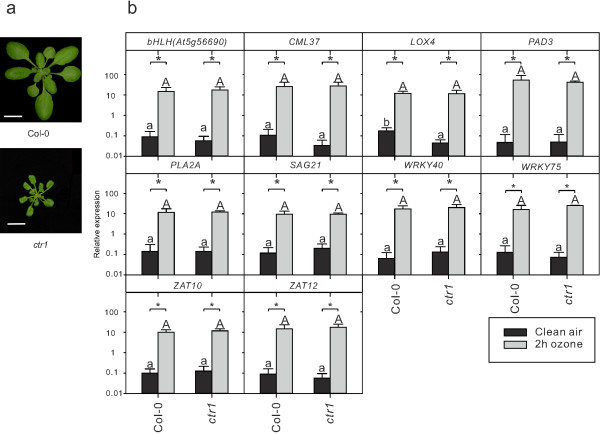
**Constitutive activation of ethylene signaling does not affect apoplastic ROS signaling. a**, Morphology of three weeks old *Arabidopsis* wildtype Col-0 and *ctr1* mutant with constitutive activation of ethylene signaling. Scale bar 1 cm. **b**, Relative expression of marker genes in clear air and after two hours of 350 nL L^-1^ O_3_ were analyzed with qPCR in *ctr1* and wildtype. Values are the mean ± SD of three biological replicates. Statistical significance (P < 0.05) was calculated on log2 transformed data by Two-way ANOVA with Tukey-test. (a, b, represent comparison of different genotypes in clean air; A, represent comparison of different genotypes in O_3_ treatment; * and n.s represent comparison between clean air and O_3_ treatment in each genotype).

The constitutive defense mutants used in Figure 2 are all characterized by having elevated SA concentration [43,61,66,67]. Another plant stress hormone, ethylene, also regulated the same set of genes as in *dnd1* (Figure 1). The *ctr1* mutant displays constitutive activation of ethylene signaling [93] and is a dwarf similar to *dnd1* (Figure 9a). However, no differences were seen between Col-0 and *ctr1* in control conditions, or after O_3_ treatment for the marker genes tested in qPCR (Figure 9b). We conclude that constitutive activation of ethylene signaling does not interfere with apoplastic ROS signaling.

## Discussion

**Figure 10:**
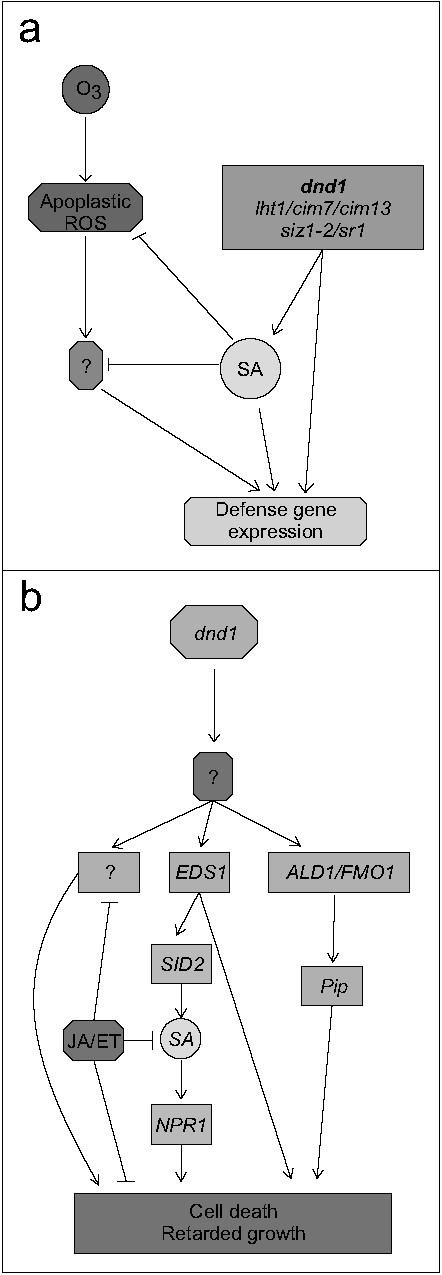
**The signaling network for interactions among SA, JA, ethylene and ROS that modulate defense activation or development of cell death. a**, SA inhibits apoplastic ROS signaling and expression of defense genes. Mutants with elevated SA accumulation display constitutive expression of defense related genes and an attenuated O_3_ gene expression response. Simultaneously abolishing SA dependent and independent signaling components (ALD1, SID2, EDS1) in *dnd1* restore its response to ozone. Moreover, pretreatment of wildtype with SA leads to a reduced response to ozone. **b**, SA-dependent and SA-independent signaling components synergistically regulate development of cell death. The exact function of DND1 function in cell death still remains to be determined, but it is likely that high SA concentration in this mutant contributes to cell death. Consequently, a mutation in SID2 can significantly reduce the amount of SA accumulation and cell death in *dnd1*. ALD1 and FMO1 are required for Pip induced SAR and SA accumulation in systemic tissues and appears to synergistically regulate cell death and defense response with SA. Moreover, EDS1 affects the onset of SA synthesis and can also directly regulate cell death. Abolishing JA in *dnd1sid2* double mutant strengthened cell death suggested that there is the anti-death regulatory function of JA signaling either through JA-SA interaction or unknown signaling components.

Antagonistic interaction between plant hormone signaling pathways is frequently observed, exemplified by SA-JA in pathogen responses and cytokinin-auxin in root and shoot development [94]. In this study we have explored another antagonistic interaction, the attenuation of apoplastic ROS signaling by SA at the level of gene expression. Several lines of evidence led to this conclusion: mutants that constitutively accumulate higher concentration of SA had a dampened response to O_3_ for several different marker genes (Figure 2b) and pre-treatment of plants with SA led to attenuation of gene expression after a subsequent O_3_ treatment (Figure 3). Conversely, increased O_3_-induced expression of SA marker genes was observed in the plants with low endogenous SA levels (Figure 4). However, it is also clear that SA alone does not fully explain why lesion mimic mutants such as *dnd1* have attenuated O_3_ responses. Blocking SA biosynthesis by introducing the *sid2* mutation into *dnd1* could only partially restore a wildtype gene expression response to O_3_ (Figure 6). Instead introduction of an additional mutation in *EDS1* or *ALD1*, giving the triple mutants *dnd1sid2ald1* and *dnd1sid2eds1*, brought back the gene expression pattern to the level of the Col-0 wildtype (Figure 8). Thus, the attenuated O_3_ response in *dnd1* is due to the inhibition on apoplastic ROS signaling by both SA dependent and independent signaling. Furthermore, two other defense hormones ethylene and JA did not appear to play any major role in this attenuation of apoplastic ROS signaling since *ctr1* had wildtype response to O_3_ and *dnd1aos* had a similar response as *dnd1* (Figsures 8 and 9b). The marker genes selected for qPCR were chosen for their O_3_ induction, however, the expression in single mutants *sid2* and *ald1* indicated that especially *PHYTOALEXIN DEFICIENT3* (*PAD3*) and *WRKY75* required a basal amount of SA to reach normal expression levels (Figures 6 and 8). In contrast *ZAT10* and *ZAT12* were not sensitive to background SA (Figure 6). The attenuation of apoplastic ROS signaling by SA was valid across all marker genes tested and highlights the robustness of the response (Figures 3 and 10a).

Treatment of Arabidopsis with flg22 rapidly activates an apoplastic RBOH-dependent ROS burst [95], and in turn induces SA related genes, including *SID2*, *PR1* and *NPR1* (*NON-EXPRESSOR OF PR GENES1*) [69,96,97]. Meta-analysis of microarray data shows that 4 hours flg22 treatment triggers similar changes in gene expression as elicited by an apoplastic ROS burst with O_3_[13], implying that flg22 and O_3_ induce similar apoplastic ROS signals. Consistent with our findings that *dnd1* has an attenuated response to O_3_ (Figure 2b), flg22 triggered oxidative burst was significantly reduced in *dnd1*[23]. Flg22 treatment can reduce SA induced changes in gene expression [98], the same type of interaction observed between apoplastic ROS and SA described in this study (Figure 3). This suggests that the effect of flg22 on SA mediated gene expression could be mediated via an apoplastic ROS burst.

If the flg22-SA and apoplastic ROS-SA antagonisms are two aspects of the same biological phenomenon, what benefit does this antagonism provide to the plant? Activating plant defenses is costly to the plant, and given the large number of potential biotic and abiotic challenges that a plant could face, it has a clear need to prioritize which challenge should be given the highest priority. This forms the basis for SA-JA antagonism where defense against insects and necrotrophic fungi rely on the JA branch and defense against biotrophic pathogens on the SA branch. The apoplastic ROS burst could have different sources, either locally from e.g. activation of RBOH or cell wall peroxidases by an invading pathogen or from systemic signaling, the so called ROS wave. The ROS wave is mediated by RBOHD generated ROS production and its ability to travel along the plant suggests that it could act as a systemic signal in response to various environmental stimuli [99]. Thus, there could be a situation where one part of the plant has already initiated SA mediated defenses due to e.g. pathogen attack, would subsequently be the recipient of the ROS wave from another part of the plant. In this situation it might be preferable to execute the already initiated local defense program and put lower emphasis on the systemic ROS signal. This might reveal itself as the antagonism by SA on apoplastic ROS signaling observed in this study and could be a beneficial way for the plant to respond and prioritize between different environmental stimuli.

Lesion mimic mutants have been crucial to identify various regulators of cell death, including the role of hormones and ROS [37,100]. As a lesion mimic mutant, *dnd1* also contributes to the study of e.g. the potential role of Ca^2+^ in cell death regulation. The extensive double mutant collection generated in this work to study the role of apoplastic ROS signaling also allow the dissection of signaling pathways involved in regulation of cell death in *dnd1*. Of the 22 double mutants and seven triple mutants generated, many of them did not alter the extent of cell death, thus excluding a role for MAP kinases, G-proteins and several transcription factors in execution of cell death in *dnd1* (Table 1). However there were several informative mutant combinations mainly related to ethylene, JA, SA and SA-related processes. The ethylene mutants (*etr1-1, ein2*) and JA biosynthesis mutant (*aos*) enhanced growth defects of *dnd1*, but did not alter the extent of cell death, implicating that these hormonal signal pathways in the *dnd1* background are not strictly required for cell death execution. In contrast, *ein2* enhances cell death in the lesion mimic double mutant *syp121syp122*[101] and the JA insensitive *coi1 (coronatine insensitive1)* enhances the lesions in *hrl1* (*hypersensitive response-like lesions1*) [102,103]. Thus, the requirements for ethylene and JA in cell death regulation appear to be context dependent.

SA is a crucial regulator of cell death shown by introducing the SA deficient mutant *sid2* or a bacterial salicylate hydroxylase (*NahG*) into several lesion mimic mutants, including *acd6*, *atg5*, and *dnd1*[37,46,104]. These observations indicate that biosynthesis of SA via ICS1 acts as a central hub of a SA inducing cell death program. However, SA depletion by introducing *sid2* could only partially relieve the cell death in *dnd1*[59]. Several other mutations which are typically associated with or acting in parallel with SA also partially reduced cell death in *dnd1* and included *eds1, pad4, ald1* and *fmo1* (Table 1; Additional file 2). Furthermore, substantially reduced cell death and improved growth was observed in triple mutants with *ald1, eds1, fmo1* or *pad4* in the *dnd1sid2* background (Figure 5 and Additional file 2). FMO1 is a suggested positive regulator of cell death [105]. ALD1 is associated with biosynthesis of Pip (a non-protein amino acid pipecolic acid, a product of lysine degradation). Endogenous Pip is a regulator of SAR (systemic acquired resistance) and contributes to defense and SA signal amplification [49]. Since cell death was further reduced in *dnd1ald1sid2* as compared to the double mutants *dnd1sid2* and *dnd1ald1*, this indicates that SA and the lysine catabolite Pip function synergistically in regulating cell death (Figure 10b). EDS1 and PAD4 are interacting proteins that play multiple roles in plant defenses, including regulation of cell death and amplification of transcriptional responses [106]. Expression of *EDS1* is negatively regulated by *CAMTA3*/*SR1* (a CaM binding transcription factor) [86]. Mutation of *CAMTA3*/*SR1* in *dnd1* background resulted in enhanced cell death (Figure 5), possibly a result of increased *EDS1* signaling and increased SA production in *dnd1sr1*.

Extensive double and triple mutant analysis to find regulators of cell death has been done in the background of *acd6* and *syp121syp122*[46,101]. ACD6 encodes a plasma membrane protein with a cytoplasmic ankyrin repeat motif, but how this protein migh activate cell death is unknown. The *syp121syp122* double mutant lacks two syntaxin proteins which are part of the SNARE machinery, controlling vesicle traffic and bulk transport of cargo in cells. Despite the different biological processes impaired in *dnd1*, *acd6* and *syp121syp122*, exactly the same regulators were found to be the crucial in all three lesion mimic mutants, and implicate SA biosynthesis (via SID2), in combination with EDS1, PAD4, ALD1 or FMO1 as the major pathway towards cell death. Furthermore, other double mutants between various lesion mimic mutants and i.e. *sid2* or *eds1* show the same suppression of cell death and include *acd11*[42], *lsd1*[107], *ssi2*[48] and *lht1*[43]. Thus in contrast to the context dependence of JA or ethylene for cell death execution, the requirement for SA and EDS1 appears more universal.

Future research should focus on how EDS1, PAD4, ALD1 and FMO1 interact with SA to regulate cell death. It is unlikely that low SA accumulation on its own would be sufficient to fully prevent cell death [46,48,59]. EDS1 shuttles between the cytoplasm and nucleus, where nuclear EDS1 localization regulates defense gene expression [108] and cytosolic EDS1 regulates cell death [106]. However, SA might be more likely to execute its function through changes in gene expression. Thus one potential explanation for the full suppression of cell death in lesion mimics when both *sid2* and *eds1* are mutated could be that both nuclear and cytosolic regulators of cell death are removed. ALD1-dependent Pip accumulation in systemic leaves during SAR is dependent on FMO1, indicating that there is possible signal amplification loop between Pip, ALD1, FMO1 and SA [49,109]. All together, we propose a signaling network where *ALD1*, *EDS1*, and *FMO1* work synergistically with SA to induce cell death in lesion mimic mutants (Figure 10b).

## Conclusions

In summary, we have identified an antagonistic relationship between SA and apoplastic ROS signaling that regulate defense gene expression in plants. This mechanism is likely timing and context dependent. Furthermore, identification of regulatory components required for execution of cell death in *dnd1* reinforces the crucial role of SA, ALD1 and EDS1 in cell death regulation. How the altered cytosolic Ca^2+^ transport in *dnd1* connects to downstream signaling pathways will require more studies and may include a recently identified *dnd1* suppressor mutant, *repressor of defense no death1* (*rdd1*) [110].

## Methods

### Plant materials and growth conditions

Mutant seeds were obtained from the Nottingham Arabidopsis Stock Centre (NASC; http://arabidopsis.info/) or were gifts from Dr Günter Brader (*wrky70*), Dr Hans Thordal-Christensen (*ald1*, *fmo1*), Dr Heribert Hirt (*mpk3*, *mpk6*), Dr. Jeff Dangl *(rar1-21*), Dr. Alan Jones (*gpa1*, *agb1*), Dr. Bonnie Bartel (*ibr5*), Dr Miguel Torres (*rbohD*, *rbohF*) and Dr. Roberto Solano (*jin1*). Wild type Arabidopsis accession Columbia-0 (Col-0) was used as control plant for all experiments. Double and triple mutants were constructed using *dnd1* as pollen acceptor. All mutants were in the Col-0 background, double and triple mutants were screened for the visible *dnd1* mutant phenotype (dwarf, curly leaves, and early senescence) and subsequently genotyped using PCR-based CAPS, dCAPS and T-DNA markers (see Additional file 3). The homozygosity of all double and triple mutants was confirmed in F3 or F4 generations.

Seeds were sown on germination medium containing ½ Murashige and Skoog (MS) and 0.4% phyto gel, stratified for three days, the plates were placed at 22°C/19°C under a 12-h light/12-h dark cycle for one week. Subsequently, one week old plants were transplanted into 1:1 peat: vermiculite mixture, five seedlings per pot (8 × 8 cm), grown at 22°C/19°C, and relative humidity of 70%/90%, under a 12-h light/12-h dark cycle for two weeks. All plants were grown in controlled environment growth chambers (Weiss Bio1300; Weiss Gallenkamp). Three weeks old plants were used for all experiments. Plants for O_3_ treatment and clean air control were randomized and grown side by side in identical environment.

### Ozone and SA treatment

O_3_ treatment was started at 9 am. Three weeks old plants were exposed with 350 nL L^-1^ ozone for two hours. To study the role of SA, Col-0 was treated with 0.3mM and 1 mM SA for 24-hr before ozone exposure. All samples were harvested in parallel from ozone treated and clean air control after the onset of ozone treatment, and immediately shock-frozen in liquid nitrogen.

### Determination of cell death

Three and five week old plants grown in clean air were used for trypan blue staining. From three rosettes per genotype and staining, one fully expanded and representative leaf (not the oldest leaf) was used for figures. The experiment was repeated at least three times per genotype. Trypan blue stain was performed as previously described in [111].

### RNA isolation

5-15 plants per genotype from control or O_3_ treatment were pooled, frozen in liquid nitrogen and stored at -80°C. Total RNA was extracted using GeneJet Plant RNA purification Mini Kit (Fermentas, now part of Thermo Scientific).

### Microarray analysis

RNA was isolated from three to four week old Col-0 and *dnd1* plants. RNA samples from six biological replicates were used for cDNA synthesis, labeling with Cy3 and Cy5, and array hybridization was done as previously described [65]. Full experimental details and raw data are available from ArrayExpress, accession number E-MEXP-3768. The *dnd1* raw data and Affymetrix raw data were processed with robust multiarray average normalization using Bioconductor limma and affy packages in R [112,113]. Gene expression for each experiment was computed by log2-base fold changes between treatment and control, or between wild type and mutants. The processed data was discretized and clustered using Bayesian Hierarchical Clustering method, as implemented in the R package BHC [114]. Bootstrap analysis was done as previously described in [20].

Raw data from the Affymetrix ATH1-121501 platform was obtained from several data sources: NASC Arrays http://affymetrix.arabidopsis.info/link_to_iplant.shtml (BTH, NASCARRAYS-392; Senescence experiment 1, NASCARRAYS-52; Senescence experiment 2, NASCARRAYS-150; SA, NASCARRAYS-192). (ArrayExpress http://www.ebi.ac.uk/arrayexpress/ (MeJA, EATMX-13) Gene Expression Omnibus http://www.ncbi.nlm.nih.gov/geo/ (H_2_O_2_, GSE5530; Syringolin, E-MEXP-739; *csn3*, *csn4* and *csn5*, GSE9728; *lht1*, GSE19109; *mkk1mkk2*, GSE10646; *sni1*, GSE6827; *siz1*, GSE6583; SA 24 h, GSE14961; Ethylene, GSE14247; Flg22, GSE5615; *Botrytis cinerea* infection, GSE5684; *Pseudomonas syringae* ES4326, GSE18978;). Raw data for *acd11*[115] were obtained from John Munday.

### Real-time quantitative PCR analysis

Two ug of RNA was DNAseI treated and used for cDNA synthesis with RevertAid Premium Reverse Transcriptase according to the manufactures’ instructions (Fermentas, now part of Thermo Scientific). The reverse transcription reaction was diluted to a final volume of 100 ul, and 1 ul was used per PCR reaction. Quantitative PCR was performed in triplicate with EvaGreen Supermix (Solis Biodyne) on a CFX384 thermal cycler 1000 (Bio-Rad). The cycle condition was performed as previously described [116]. Three reference genes (*SAND*, *TIP41*, *YLS8*) were used for normalization. Amplification efficiency of all primer pairs were calculated through amplification of serially diluted cDNA. Primer sequences and amplification efficiency are listed in Additional file 4. Gene expression analysis was performed using qBaseplus2 (Biogazelle). At least three biological repeats per experiment were used for analysis. Statistical analysis was calculated by two-way ANOVA with Tukey-test using SigmaPlot 11.0.

## Competing interests

The authors declare that they have no competing interests.

## Authors’ contributions

MB conceived the study, participated in its design and coordination, performed microarray hybridization, participated in gene expression studies and helped to draft the manuscript. EX participated in its design, performed phenotyping and genotyping double and triple mutant, gene expression studies, cell death identification, performed data analysis, and wrote the manuscript. Both authors read and approved the final manuscript.

## Supplementary Material

Additional file 1 **Genes with significant change of expression in *dnd1 *** **compared with Col-0 identified through microarray analysis.** Click here for file

Additional file 2 **Visual phenotype of five week old *dnd1 *** **single, double, and triple mutants.** Five week old plants were used to visualize cell death with trypan blue staining. From three rosettes per genotype and staining, one fully expanded and representative leaf (not the oldest leaf) was used for figures. Click here for file

Additional file 3 Primers and restriction enzymes used for mutant genotyping. Click here for file

Additional file 4 Primers used in qPCR and amplification efficiencies. Click here for file
