# Supplementary figures and images for: Salicylic acid signaling inhibits apoplastic reactive oxygen species signaling
Source: BMC Plant Biol. 2014 Jun 4;14:155. doi: 10.1186/1471-2229-14-155 (PMC4057906; doi:10.1186/1471-2229-14-155)

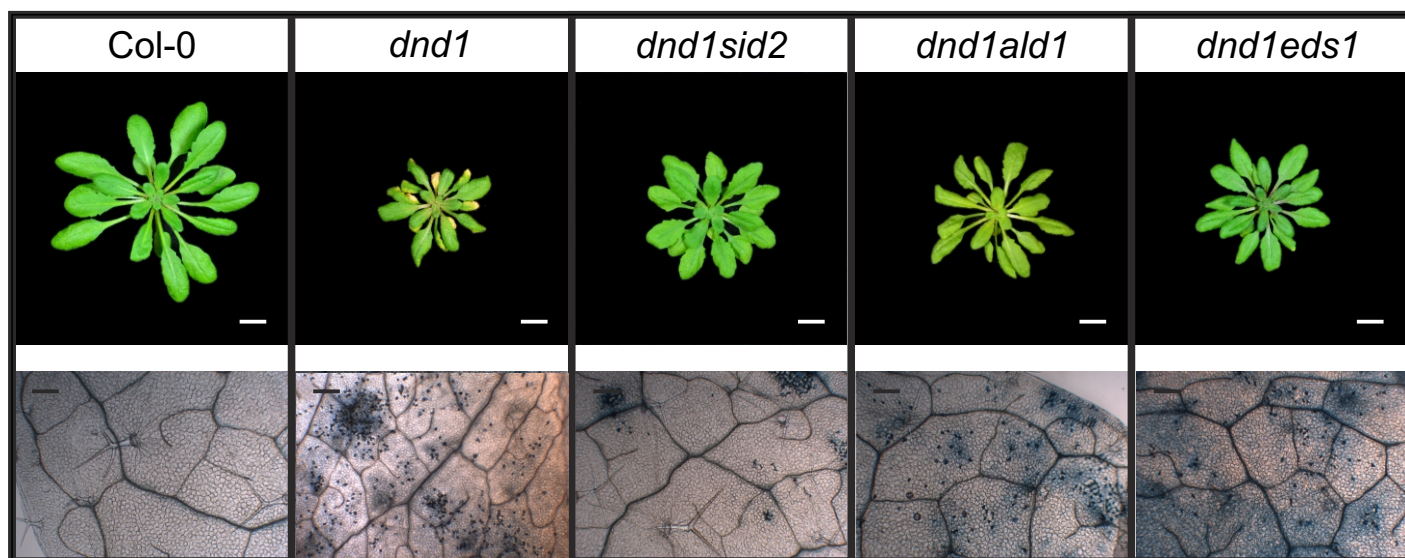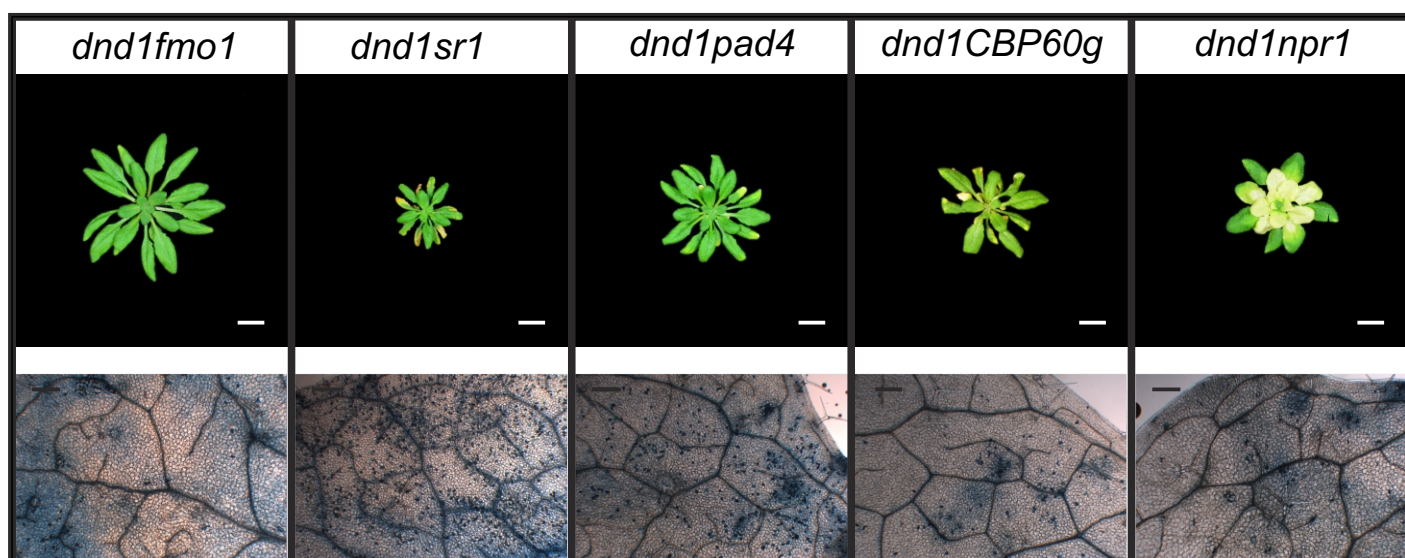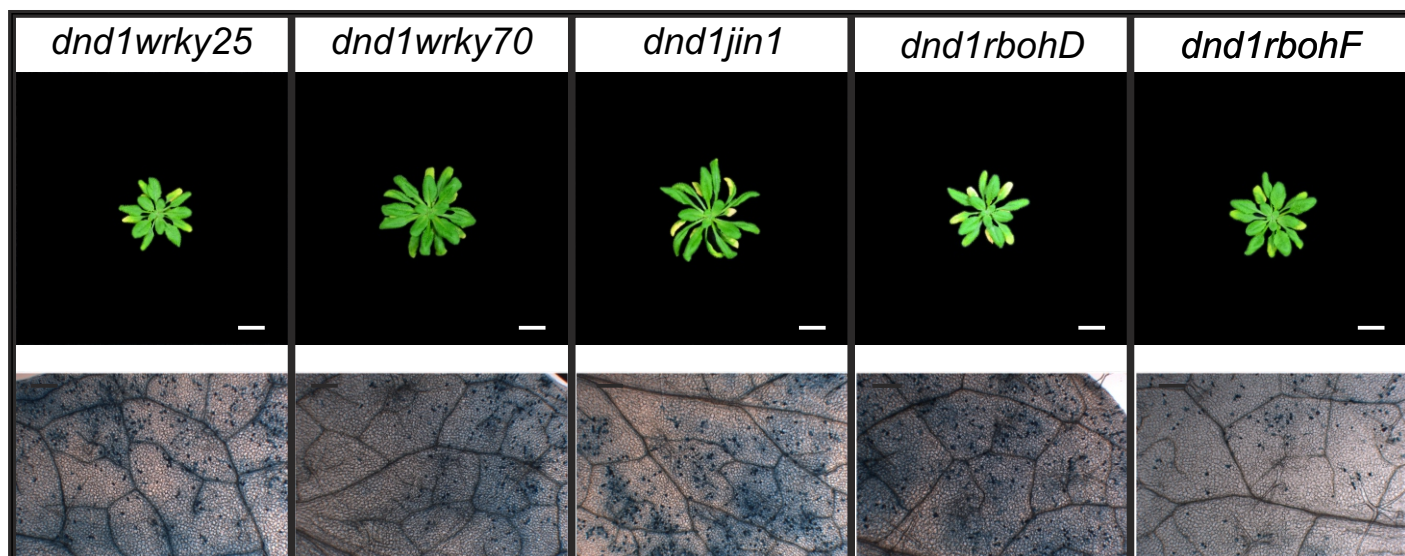

Supplement: Additional file 2 — Visual phenotype of five week old dnd1 single, double, and triple mutants. Five week old plants were used to visualize cell death with trypan blue staining. From three rosettes per genotype and staining, one fully expanded and representative leaf (not the oldest leaf) was used for figures. [file 1471-2229-14-155-S2.zip › 1758204575123856_MOESM2_ESM.pdf]

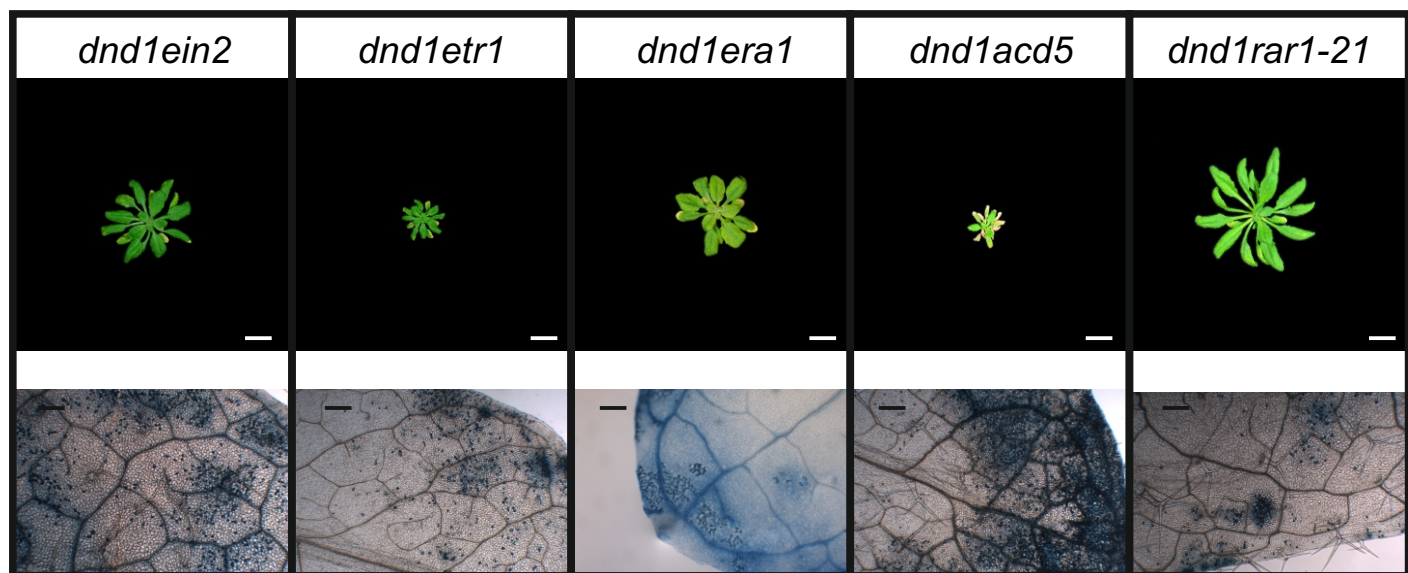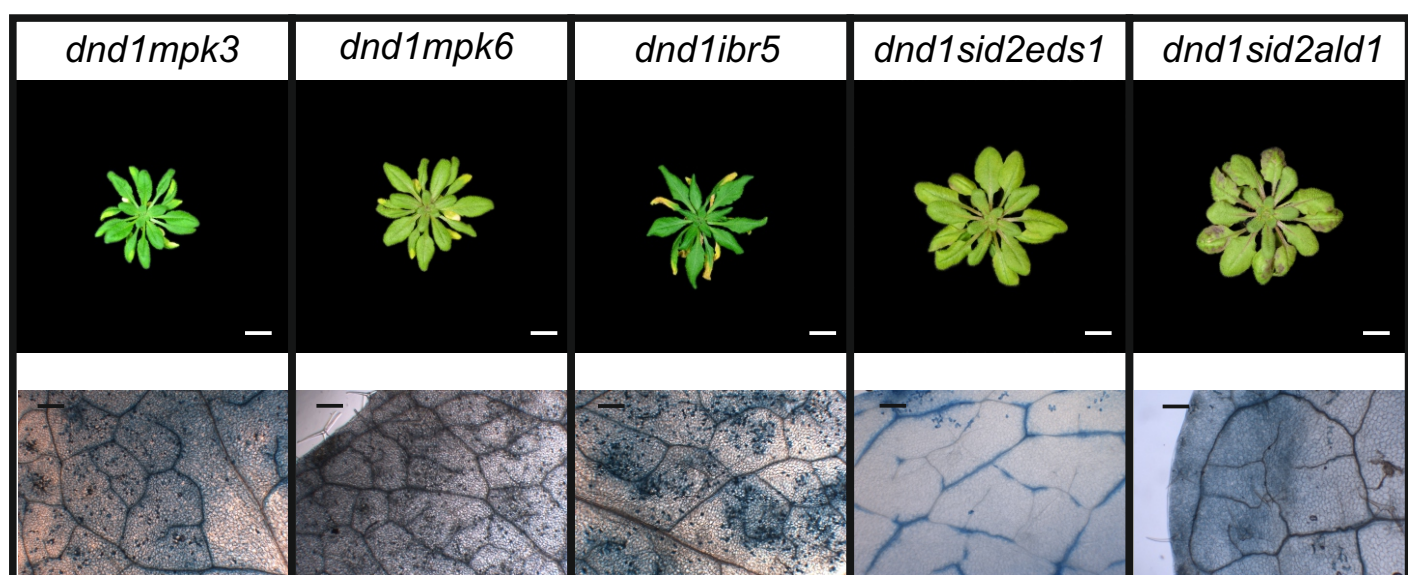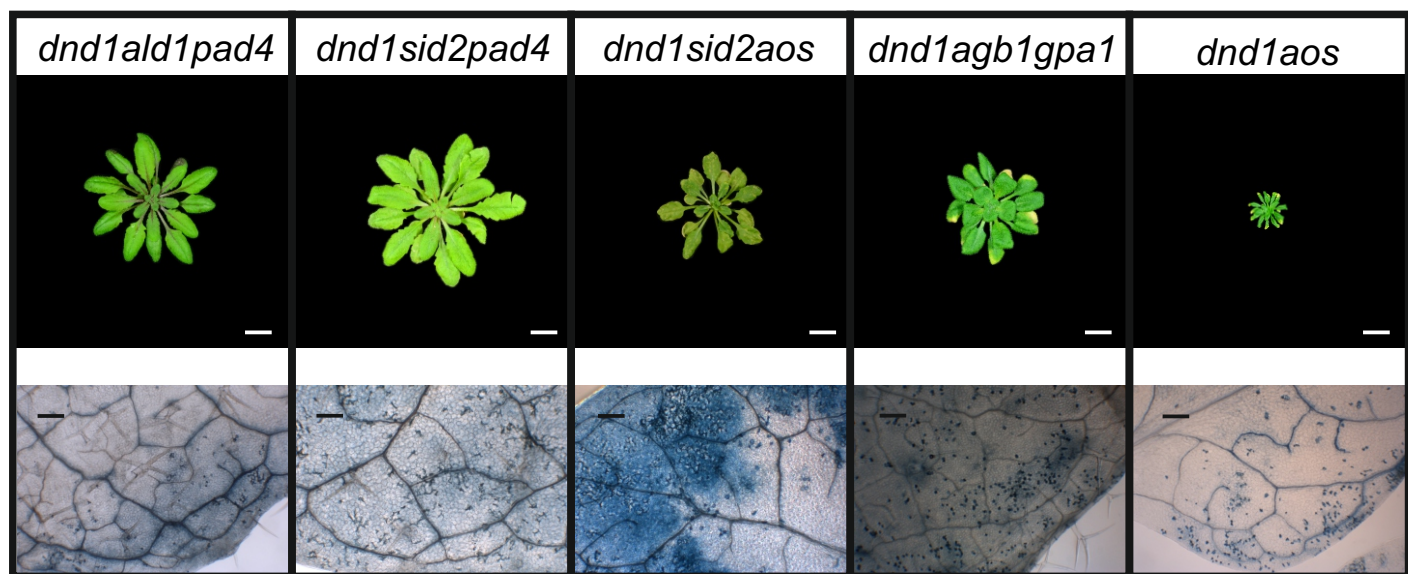

Supplement: Additional file 2 — Visual phenotype of five week old dnd1 single, double, and triple mutants. Five week old plants were used to visualize cell death with trypan blue staining. From three rosettes per genotype and staining, one fully expanded and representative leaf (not the oldest leaf) was used for figures. [file 1471-2229-14-155-S2.zip › 1758204575123856_MOESM3_ESM.pdf]
